# Beneficial Effects of Bovine Milk Exosomes in Metabolic Interorgan Cross-Talk

**DOI:** 10.3390/nu14071442

**Published:** 2022-03-30

**Authors:** Jorge García-Martínez, Íñigo M. Pérez-Castillo, Rafael Salto, José M. López-Pedrosa, Ricardo Rueda, María D. Girón

**Affiliations:** 1 Abbott Nutrition R&D, Abbott Laboratories, 18004 Granada, Spain; jorge.garciamartinez@abbott.com (J.G.-M.); inigomaria.perez@abbott.com (Í.M.P.-C.); jose.m.lopez@abbott.com (J.M.L.-P.); ricardo.rueda@abbott.com (R.R.); 2 Department of Biochemistry and Molecular Biology II, School of Pharmacy, University of Granada, Campus de Cartuja, 18071 Granada, Spain; mgiron@ugr.es

**Keywords:** milk, exosomes, extracellular vesicles, miRNA, immunity, intestinal health, bone, muscle, microbiota, neurodevelopment

## Abstract

Extracellular vesicles are membrane-enclosed secreted vesicles involved in cell-to-cell communication processes, identified in virtually all body fluids. Among extracellular vesicles, exosomes have gained increasing attention in recent years as they have unique biological origins and deliver different cargos, such as nucleic acids, proteins, and lipids, which might mediate various health processes. In particular, milk-derived exosomes are proposed as bioactive compounds of breast milk, which have been reported to resist gastric digestion and reach systemic circulation, thus being bioavailable after oral intake. In the present manuscript, we critically discuss the available evidence on the health benefits attributed to milk exosomes, and we provide an outlook for the potential future uses of these compounds. The use of milk exosomes as bioactive ingredients represents a novel avenue to explore in the context of human nutrition, and they might exert important beneficial effects at multiple levels, including but not limited to intestinal health, bone and muscle metabolism, immunity, modulation of the microbiota, growth, and development.

## 1. Background

Extracellular vesicles (EVs) were first described in the 1960s and 1970s and were considered mere cellular artifacts lacking a biological purpose [1,2]. It was not until the decade of the 1990s that EVs were attributed a role in cell-to-cell communication, thereby gaining increased attention from the scientific community [2,3]. Since then, EVs have been isolated from almost all mammalian cells and are known to be present in virtually all body fluids constituting a burgeoning field of research [4,5].

No consensus on EVs definition has been reached so far. However, EVs are generally considered as membrane-enclosed secreted vesicles that encompass various subsets of different compounds, namely exosomes, ectosomes, microvesicles, microparticles, and apoptotic bodies [6]. These different EVs subsets are characterized based on their size, biological origins, release pathways, functionality, and cargos [7]. In particular, the study of exosomes has witnessed breakthrough progress in recent times. Exosomes are typically considered as molecules sized around 10–200 nm, which originate from repeated invagination of the lipid bilayer membrane of multivesicular bodies [8,9] and can harbor a variety of different cargos and components, such as lipids, proteins, and nucleic acids, thus acting as delivery molecules [10]. Regarding their cargos, the nature of the exosomal content is heterogeneous and depends on its cellular origins and microenvironment [11]. However, the loading of cargos into the exosome is a non-random process that obeys their biological purpose [12]. Hence, given the importance of exosomes in cell-to-cell communication and their presence in diverse body tissues, a database was developed in 2009 with the endeavor of cataloging exosome cargos from all types of tissues [13]. To this date, more than 1960 proteins, 2830 miRNAs, and 1110 lipids have been reported in exosomes [14].

Cellular Origins of Exosomes

From the scientific point of view, one of the most interesting aspects of exosomes resides in their unique biogenesis process. Exosomes biosynthesis begins with the endocytosis of extracellular environment constituents by invagination of the plasma membrane along with the internalization of their associated surface proteins, which leads to the formation of an early-sorting endosome (ESE). The trans-Golgi network and the endoplasmic reticulum can contribute to the content of the ESE, which matures into late-sorting endosomes (LSE) to eventually generate multivesicular-bodies (MVBs). MVBs contain intraluminal vesicles that will constitute future exosomes once the MVB fuses with the plasma membrane [11]. In contrast, larger EVs, such as microvesicles and apoptotic bodies, originate via budding and shedding from the plasma membrane of cells. Several molecules, such as TSG101, ALIX, ESCRT, and ceramides, among others, are involved in the biogenesis of exosomes and, therefore, have been proposed as exosome (specific) markers [5,11,15]. However, these proteins might be involved in the trafficking of other vesicles, thus potentially acting as a confounding factor when conducting research in the field of exosomes [11]. Similarly, exosomes usually display certain proteins in their membranes, namely tetraspanins CD9, CD63, and CD81, which can serve as exosome biomarkers, albeit caution must be warranted as tetraspanins can be indistinctly expressed in the membrane of different vesicles [16].

Due to their biogenesis process, exosomes content resembles that of the donor cell. Thus, the characteristics of exosomes components and cargos will depend mainly on the tissue where they originate. As previously stated, exosomes have been isolated from virtually all human fluids, including plasma, saliva, urine, and breast milk [17]. In this regard, the presence of exosomes in breast milk is of particular interest, given that not only does milk constitute fundamental nutrition for the newborn but also contains bioactive compounds which have been implicated in growth, neurodevelopment, and immunomodulatory processes [18,19,20].

## 2. Materials and Methods

We comprehensively analyzed the available scientific literature on milk exosomes, including both reviews and original research articles. An extensive search was conducted in the scientific databases and search engines PubMed, ScienceDirect, and Google Scholar using relevant search terms (i.e., “exosomes”, “extracellular vesicles”, “milk”, “dairy”) in combination with Boolean operators (i.e., “AND”, “OR”, “NOT”) with no time restrictions. Additionally, the ProQuest Dialog^®^ search tool was used to screen different electronic databases and repositories such as Embase, BIOSIS Previews, and ProQuest Dissertations and Theses Professional to supplement the literature search.

## 3. Milk-Derived Exosomes

**Figure 1:**
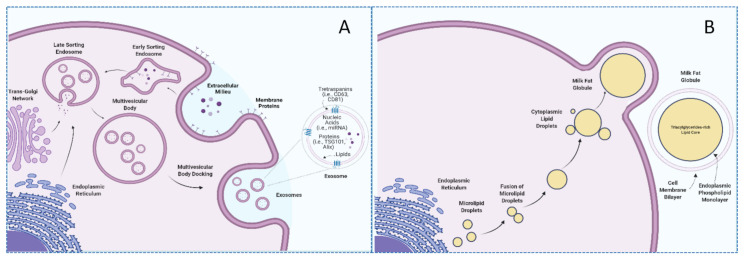
Biogenesis of milk exosomes and milk fat globules. (**A**), milk exosomes; (**B**), milk fat globules.

Milk is a nutrient-rich fluid produced by female mammals whose main purpose is to meet the nutritional requirements of the newborn. Due to its excellent nutritional profile, milk constitutes an important component of the human diet [21]. In addition to nutritional properties, milk contains a wide repertoire of bioactive compounds to which potential health benefits have been attributed [22]. In particular, different EVs, including exosomes and milk fat globules (MFG), are included among described milk bioactive compounds [23]. These vesicles have different sizes, origins, compositions, and functions. Specifically, MFG are far bigger structures (up to 20 μm) constituted by a triacylglyceride-rich core enveloped by a polar lipid trilayer [24], while exosomes are nanosized (10–200 nm), lipid bilayered vesicles with notably lower triacylglyceride content [25]. In the same fashion, lipidomic analyses have suggested that these compounds differ in the presence of phospholipids, with a higher proportion of phosphatidylserine and sphingomyelin being reported in human and bovine milk exosomes in comparison with milk fat globule membranes (MFGM) [25]. Not only lipid but also protein compositions of MFGM and exosomes display notable differences [25,26,27]. According to the BoMiProt database, more than 1300 proteins have been exclusively found in bovine milk exosomes, while 294 have been identified in MFGM [26]. These differences would arise due to their different biogenesis pathways. Whereas exosomes have endosomal origins, MFG are formed in the endoplasmic reticulum and are released into the cytosol as lipid droplets [28,29] (as illustrated in Figure 1).

### 3.1. Milk Exosomes Isolation and Characterization Methods

The isolation of milk exosomes remains a challenging process. Since extracellular vesicles are an emergent field of research, no consensus has yet been reached on isolation or characterization methods. The International Society for Extracellular Vesicles (ISEV) provided criteria and recommendations for the isolation and analysis of EVs, which can serve as guidance for the accurate reporting of exosomes studies [6,30]. Accordingly, these studies should describe in detail the methods employed to isolate and characterize exosomes and report aspects related to these processes, such as purity, size distribution, and enriched markers assessed [6]. In this regard, the methods used to isolate exosomes from different sources are also applicable to milk exosomes. However, matrix substances, such as milk caseins, which account for >80% of bovine milk proteins, can interfere with the isolation process and the lack of accuracy in separating exosomes from different constituents represents the main limitation of available techniques [31].

The most widely used method to isolate milk exosomes is differential ultracentrifugation, which is considered to be the current “gold standard” technique [31,32]. Sequential ultracentrifugation is needed to separate exosomes from other colloidal structures, and it is usually followed by an additional gradient step (i.e., sucrose gradient) [33,34]. Density gradient ultracentrifugation yields low protein contamination and high purity, but it is a time-consuming process and might be unsuited for large-scale production due to its low sample throughput and potential negative impact on exosome integrity [33,35].

Size-exclusion chromatography (SEC) has also been successfully implemented to isolate exosomes from different origins. SEC relies on the differential elution profiles of different particles running through a stationary phase depending on their size [36]. This technique has been used to isolate bovine milk exosomes improving both yield and time efficiency compared to differential ultracentrifugation [25,37]. On this subject, a method for isolating EVs using a size-exclusion chromatography column in combination with turbidimetry was developed and applied to cow milk samples [38]. SEC presents practical advantages over differential ultracentrifugation, being simpler, less expensive, and reusable [36]. Nonetheless, a recent report suggested that SEC might be incapable of differentiating exosomes from other milk components when assessing industrially processed milk samples [39]. The presence of other contaminants and different EVs subsets might compromise SEC analyses in exosomes studies [31,37]. Overall, most authors favor using a combination of methods to ensure an adequate isolation process [40]. Recently, the combination of ultracentrifugation and SEC, or tangential flow filtration (TFF), along with divalent cation chelation with EDTA, was shown to produce large amounts of pure milk EVs isolates, thus constituting a promising approach [40].

Milk caseins represent an important obstacle to the isolation of milk exosomes. Methods based on acidification have been explored to cause isoelectric precipitation of milk caseins, thus enabling their separation from other milk components [34,41]. Acidification enables faster isolation of milk exosomes and yields significantly higher amounts compared to differential ultracentrifugation [32]. Accordingly, this process has been proposed as the most suitable step to remove caseins [42]. However, some authors have reported deterioration of exosomes’ surfaces after acid treatment [34,41].

Commercial kits were introduced to the market to isolate EVs with similar results to those obtained by ultracentrifugation techniques without requiring specialized equipment [31]. Among these, exosome precipitation solutions (ExoQuick^TM^, System Biosciences, Palo Alto, CA, USA), and membrane affinity spin columns (ExoEasy^TM^, QIAGEN, Hilden, Germany) have been applied to milk extracellular vesicles [43,44,45,46,47]. These kits provide rapid EVs isolation but are susceptible to high protein contamination and can promote exosome aggregation [31,48]. Particularly, the incapacity to differentiate different EVs subsets is a common disadvantage of most available methods. Newer techniques, such as asymmetrical flow field-flow fractionation (AF4), have been shown to enable the differentiation between different EVs populations up to 1 nm increments [48,49], albeit low yield and high costs may hinder their implementation [50].

The characterization of milk exosomes is crucial to evaluating the heterogeneity of isolated preparations [6]. Diverse methods are available for this purpose. Transmission electron microscopy (TEM) and cryo-electron microscopy (Cryo-TEM) permit the characterization of exosomes by their size and distribution and are widely used in exosomes research [31,51]. In the same line, adapted high-resolution flow cytometry protocols were demonstrated to enable the characterization and quantification of milk exosomes [52]. These protocols are typically used in conjunction with TEM or cryo-TEM in milk exosome studies [53,54]. Other techniques, such as nanoparticle tracking analysis (NTA), which is usually used in combination with fluorescence detection, can effectively measure particle size, distribution, and concentration in liquid media [31,55] and are applicable to validate the isolation of milk exosomes [56]. Similarly, dynamic light scattering (DLS) has been used to measure the size distribution of milk exosomes [57,58]. However, it is possible that DLS cannot differentiate populations with different sizes, being useful only to evaluate populations of monodisperse samples [29,31]. Large-scale and cost-effective isolation of milk exosomes constitutes a bottleneck for their successful implementation as bioactive ingredients and methods available so far might not be suited for this purpose [59].

As commented, milk exosomes can be characterized based on enriched marker proteins such as tetraspanins (i.e., CD9, CD63), trafficking molecules (i.e., TSG101, ALIX), and chaperones (i.e., HSC70, HSP60) [42,60]. Detection of these markers by Western-Blot is customary in milk exosomes studies [43,61,62]. Finally, the combination of specific antibodies and atomic force microscopy (AFM) provides better resolution than other imaging techniques but requires specialized equipment and has limited throughput [31,42]. Remarkably, given the limitations of existing techniques, the combination of different methods, including microscopy, flow cytometry, and Western blotting, should be considered to ensure an adequate characterization of isolated exosomes [60].

### 3.2. Milk Exosomes Composition and Cargos

Milk exosomes harbor various cargos with potential roles in intercellular communication, metabolism, and nutrition. Beyond tetraspanins, exosomes can contain many different proteins, including membrane transport proteins, cell adhesion proteins, signaling proteins, enzymes, cytoskeletal proteins, and chaperones [15,63]. Furthermore, exosomes from bovine colostrum were shown to be significantly enriched in proteins implicated in the growth and immune response [64]. Among the most consistently reported proteins of milk exosomes are the lactadherin [65], a glycoprotein that has been observed to play a role in maintaining the intestinal epithelium [66,67]. Besides, bovine milk exosomes were shown to carry bioactive transforming growth factor-β1 (TGF-β1), a cytokine with important immunomodulatory properties [68,69].

Contrary to proteins, the lipid composition of exosomes remains largely unexplored. Lipidomic analyses of milk-derived exosomes have revealed that phosphatidylcholine (PC), phosphatidylserine (PS), phosphatidylethanolamine (PE), and sphingomyelin (SM) are among the most enriched lipids found in these vesicles [25,70,71]. PC has been proposed to ameliorate inflammation via the gut–brain axis in vivo [72]. Likewise, PE might present hypocholesterolemic effects reducing intestinal lipids absorption and SM has been proposed to promote myelin formation and brain neuroplasticity, thus potentially playing a beneficial role in infant neurodevelopment [73,74]. Evidence from human trials attributes a positive effect to MFGM lipids in growth, intestinal health, and infant neurodevelopment [75]. These effects are usually attributed to polar lipids, which are also abundantly expressed in milk exosomes [70]. However, research on the beneficial effects of the lipidic fraction of milk exosomes is lacking and constitutes an interesting avenue to explore [75].

**Figure 2:**
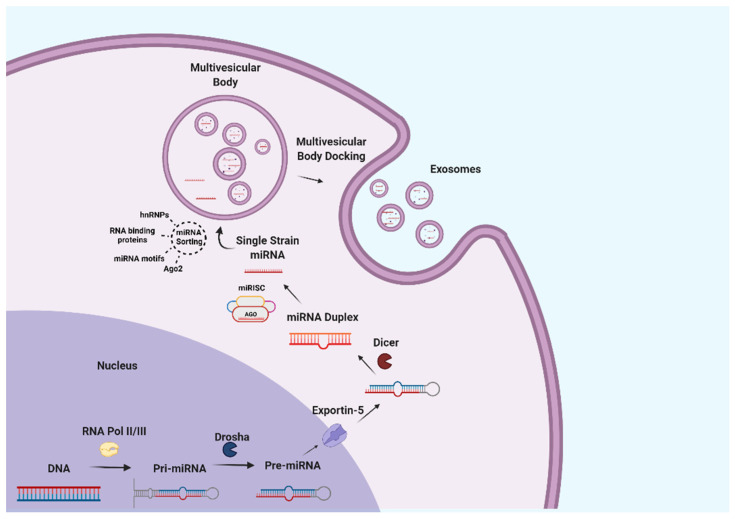
MiRNA synthesis and loading into milk exosomes.

Among exosome components, nucleic acids have attracted the greatest interest due to their biological effects on regulating metabolic processes [5]. Different nucleic acids have been described in milk exosomes, including DNA, mRNA, miRNA, circular RNA, long non-coding RNA, etc. In particular, milk represents one of the richest sources of miRNA [32,76]. MiRNAs are defined as 17–24-nucleotide small noncoding RNA fragments which are responsible for post-transcriptional gene silencing by binding to regions of target mRNAs [77]. In animals, miRNA genes are transcribed to pri-miRNAs that are processed by the nuclear RNase III Drosha, and the resulting intermediates are exported out of the nucleus via exportin-5 to be later cleaved by the RNase III Dicer into a miRNA duplex [78]. The resulting miRNA duplex associates with Argonaute (Ago) proteins, which are part of the RNA-induced silencing complex (miRISC), leading to the removal of the passenger strand and the final maturation of the miRNA [79]. However, miRNAs can also be loaded into exosomes in a completely independent pathway which might implicate multiple mechanisms, namely recognition by hnRNPA2B1 and hnRNPA1 RNA-binding proteins [80,81], an affinity for different cell membrane lipids [82], recognition of specific RNA motifs and configurations, etc. [83]. These processes are illustrated in Figure 2.

Including milk, biological fluids constitute an aggressive environment for miRNA viability. Exosomes protect miRNAs against low pH and RNases, thus enabling their delivery to target cells [35,84,85]. Furthermore, exosomes have been shown to confer protection against the adverse conditions present in the digestive tract, which suggests that the oral intake of these compounds facilitates the availability of their cargos [86,87,88]. Besides protecting their cargos, glycoproteins displayed in milk exosomes facilitate their recognition and uptake by the target cell receptors [70,89].

Interestingly, milk exosomal miRNAs are highly conserved across mammalian species, including humans [90]. A remarkable number of these milk miRNAs are considered to be related to the immune system [91,92]. Particularly, miR-155 has been shown to regulate T cells development and homeostasis [93,94]. Other miRNAs, such as Let-7c, miR-17, miR-30, miR-92, miR-148a, and miR-223, have been linked to the regulation of different populations of immune cells and inflammatory processes [95,96]. Synapse localization is strongly enriched among genes targeted by miRNAs abundantly expressed in milk exosomes, which might positively affect brain development during early life [97]. MiR-181a-5p has been consistently reported to be among the most highly expressed miRNAs in milk exosomes [98,99], and it is considered an antiatherogenic miRNA that downregulates NF-κB activation and vascular inflammation [100,101]. Hence, milk exosomes may promote the transfer of miRNAs from the mother to the offspring, playing a role in the early development of the immune system and mediating different positive health effects [102].

### 3.3. Bioavailability, Bioaccessibility, and Bioactivity of Milk Exosomes

The capability of milk exosomes to exert biological effects relies on their bioaccessibility and bioavailability after oral intake [103]. Bioaccessibility would consist of the ability of the exosomes and their cargos to survive gastrointestinal digestion. As already mentioned, strong evidence provided by in vitro human digestion models supports that milk exosomes resist gastrointestinal/pancreatic digestion processes [99,104,105,106]. This fact is even more important in infants, given their lower stomach acidity. In this sense, the free synthetic miRNAs that are added to milk are rapidly degraded by an acidic environment and RNases, while disruption of exosomes by ultrasonication releases miRNA content, thus leading to similar effects [107]. Milk exosomes not only protect miRNAs from low pH and RNases but may also exert a protective effect on cargo proteins from proteases [106].

Storage conditions, freezing, and thawing can compromise exosomes stability, negatively influencing their count in milk samples [108,109]. Nonetheless, some studies have observed that exosomes are very stable in harsh conditions. In this sense, it has been shown that exosomes contained in commercial bovine milk harbor miRNAs and immunoregulating proteins such as TFG-β after pasteurization [68]. However, different reports suggest that common industrial processing might compromise commercial milk exosome integrity, thus drastically reducing their count in products such as UHT milk or infant formulas [53]. Similarly, dairy products subjected to fermentation processes and consumer handling (i.e., microwaves) may show a significant decrease in exosome protein and miRNA content [107,110]. Processes such as fermentation may induce exosome lysis and increase RNase levels due to microbial production [107]. In the same line, infant formulas are also subjected to harsh conditions during their production, and thus, the addition of exosomes as an ingredient to resemble the characteristics of human milk must overcome challenges related to industrial processing and storage [111]. These aspects are even more relevant in hydrolyzed formulas, which are subjected to more severe processing conditions [112].

Bioavailability implies gastrointestinal absorption and the subsequent presence in the systemic circulation [103]. Studies evaluating the effects of gastrointestinal digestion on exosomes have also analyzed the uptake of exosomes and their cargos by human intestinal cells in vitro, yielding positive results [99,105]. Furthermore, studies conducted in humans have observed increased concentrations of certain miRNAs in blood mononuclear cells after the consumption of milk [113]. This fact has predisposed authors to evaluate the potential of milk-derived exosomes as carriers to facilitate oral drug administration [114]. Notably, Manca et al. evaluated the bioavailability of fluorescently labeled bovine milk exosomes and their labeled miRNAs in mice, observing that both exosomes and miRNAs were bioavailable after exosomes oral intake and intravenous administration, and were accumulated in different organs, although the distribution of miRNAs showed a unique profile [87]. Moreover, studies have reported that bovine milk exosomes are assimilated by gut bacteria in mice, thus modulating gut microbiota, suggesting that exosomes are involved in a potential cross-talk between animals and bacteria of different species [115,116]. Regarding internalization mechanisms, intestinal cells uptake exosomes through a glycoprotein-mediated endocytosis process. Accordingly, eliminating glycoproteins using proteinase K or trypsin in human and rat intestinal cells leads to a significant decrease in exosome uptake [117]. Similar results are obtained when intestinal cells are treated with endocytosis inhibitors and carbohydrate competitors [117]. Different cells can uptake exosomes by a variety of proposed mechanisms, including clathrin-dependent endocytosis, caveolin-mediated uptake, micropinocytosis, phagocytosis, etc. [118] In this regard, a recent study reported that milk exosomes are phagocyted by mice macrophages through a process mediated by class A scavenger receptors, which is important when assessing their potential as delivery vehicles [119]. Overall, the uptake mechanisms depend on the exosomes source and the type of recipient cell, and several mechanisms may co-exist [8].

**Figure 3:**
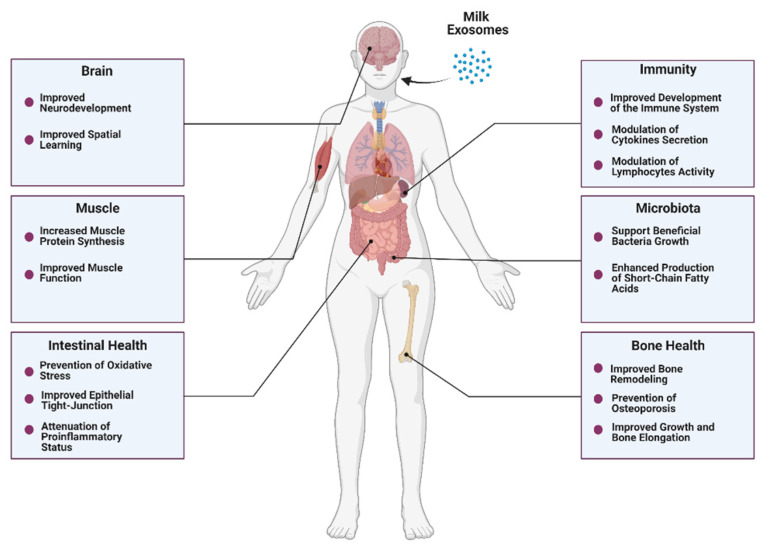
Potential beneficial effects of milk exosomes on overall health.

As discussed, milk exosomes carry proteins, lipids, and nucleic acids with the potential to regulate immunity, growth, and development [103]. The bioavailability and bioaccessibility of milk exosomes modulate their bioactivity, defined as their capacity to exert functional effects. Remarkably, current scientific evidence suggests that milk exosomes can survive harsh conditions and be bioavailable after oral ingestion. Henceforth, it seems reasonable to evaluate the beneficial effects that these bioactive compounds can mediate in health and metabolism when orally consumed (Figure 3).

## 4. Beneficial Effects of Bovine Milk Exosomes on Overall Health

### 4.1. Intestinal Health

A growing body of scientific evidence indicates that milk exosomes survive digestion processes and are assimilated by intestinal cells via endocytosis, being bioavailable systemically [87,88,99,117,120]. Nonetheless, milk exosomes can also elicit direct effects on intestinal cells when taken up. In this regard, Martin et al. reported that human milk exosomes (HME) exerted a protective effect against H_2_O_2_-induced oxidative stress in intestinal epithelial cells [121]. Ensuing studies endeavored to elucidate the underlying mechanism of action. On this subject, Dong et al. exposed intestinal stem cells to H_2_O_2_, documenting that the addition of HME led to increased cell viability, which was proposed to be mediated by the upregulation of the highly conserved Wnt/β-catenin axis [122]. Hence, HME might exert beneficial effects on intestinal oxidative stress, which is a key feature of necrotizing enterocolitis (NEC) and intestinal bowel disease (IBD) [123]. Briefly, NEC is a severe intestinal pathology that develops during infancy, whereas IBD is a group of adult chronic disorders, including ulcerative colitis and Crohn’s disease, characterized by inflammatory processes [124]. However, both NEC and IBD share common pathological features, namely compromised intestinal tight junctions, reduced mucosa layer, and increased susceptibility to bacterial components, such as lipopolysaccharides (LPS) [125], and milk exosomes have been reported to convey beneficial effects on various of these aspects.

Both raw and pasteurized human milk exosomes have been shown to reduce the injury caused by hypoxia and LPS in mouse organoids while attenuating the expression of proinflammatory IL-6 in vitro [62]. Similarly, gavage administration of these exosomes to a mouse NEC model led to reduced mucosal injury and inflammation and an increased number of goblet cells and mucosa production regardless of the pasteurization process [62]. Moreover, He et al. evaluated the effects of HME obtained from mothers who delivered term preterm infants on in vivo and in vitro NEC pathological scores. They observed that HME protected against the LPS insult and increased the expression of epithelial tight-junction proteins. Regarding animal models, pretreatment with HME led to reduced intestinal mucosal damage, lower levels of IL-6 and TNF-α, and increased expression of epithelial tight-junction proteins. These effects were independent of the delivery status [126]. Other authors have reported similar effects of HME on the incidence and severity of NEC in rat models [70,127]. In particular, Chen et al. observed that HME isolated from women who gave birth to term or preterm newborns exerted a protective effect on NEC by decreasing the severity of the injury and stimulating the proliferation and migration of intestinal epithelial cells independently of the delivery status [70]. The authors further analyzed the lipidomic profile of HME, finding that term and preterm milk exosomes displayed almost identical lipid compositions. Furthermore, bioinformatic analyses suggested that HME’s most abundant lipids were related to the ERK/MAPK pathway, which is proposed to mediate LPS injury in intestinal cells [70]. Therefore, HME not only reduces oxidative stress but also positively contributes to intestinal epithelial integrity while decreasing intestinal inflammation in NEC models.

In the light of these studies, it can be proposed that exosomes may confer therapeutic properties to human milk in NEC situations. Notably, feeding human breast milk is demonstrated to reduce the incidence of NEC compared to formula feeding [128]. However, mothers delivering preterm and low birth weight infants may be unable to initiate milk expression, thus making donor human milk necessary to ensure adequate lactation [129]. In this context, some authors have evaluated the role that milk exosomes obtained from different animal sources play in NEC as an alternative to human milk. The concomitant administration of bovine milk exosomes to NEC-induced mice was shown to protect against intestinal injury, increase the number of goblet cells, and improve endoplasmic reticulum function in vivo [86]. Similarly, bovine milk exosomes were demonstrated to induce cell proliferation, protect against oxidative stress caused by H_2_O_2_ while inhibiting Nrf2 and H01 gene expression, and improve purine nucleotide catabolism and energy status in rat intestinal crypt epithelial cells [110,130,131]. In the same line, milk exosomes originating from different species, including mice, pigs, and yak, have been demonstrated to exert beneficial effects on the features of NEC [45,132,133,134]. The addition of different nutritive compounds to infant formulas to prevent NEC development has been shown to be largely unsuccessful, and further insights into which bioactive human milk compounds are responsible for its effects on NEC prevention are required [135]. In the light of the literature, the effects of bovine milk exosomes on NEC features might resemble those of HME, thus constituting a promising new ingredient for infant formulas to address the development and treatment of NEC.

Regarding IBD, some authors have evaluated the role that milk-derived exosomes play in the characteristics of these intestinal disorders denoted by inflammatory phenotypes. Among them, Wu et al. observed that the reduction of bovine milk exosomes and their miRNA content in diets administered to Mdr1a knockout mice led to exacerbated IBD symptoms compared to exosome/miRNA-sufficient diets [136]. An ensuing pilot study conducted in a different genetic mouse model of ulcerative colitis observed that the administration of bovine milk exosomes improved the macroscopic colitis histopathological scores of treated mice compared to control [137]. Benmoussa et al. evaluated the administration of milk exosomes on colitis outcomes in a dextran sodium sulfate (DSS) -induced colitis mouse model. The authors concluded that milk exosomes partially restored intestinal impermeability, recovered mucin secretion, improved histology scores, reduced colon shortening, and prevented weight loss. In particular, milk exosomes were shown to downregulate the expression of several colitis-associated microRNAs, namely miR-21, miR-29b, and miR-125b [138]. Likewise, Reif et al. reported that the oral administration of exosomes isolated from cow and human milk exerted similar positive effects attenuating the severity of colitis symptoms, reducing IL-6 and TNF-α expression, and downregulating DNMT1 and DNMT3 methyltransferases in DSS-induced colitis mice [69]. On this subject, a recent study conducted by Tong et al. provided further evidence on the beneficial effects of bovine milk exosomes on wide aspects of ulcerative colitis. The authors observed that the treatment of RAW264.7 cells with different concentrations of bovine milk exosomes inhibited inflammatory responses mediated by TLR4-NF-κB and NLRP3 pathways. These results were replicated in vivo in a mouse model of ulcerative colitis. Moreover, the administration of bovine milk exosomes improved wide aspects of cytokine homeostasis, immune response, and modestly improved gut microbiota profile [139]. Thus, bovine milk exosomes constitute a promising therapeutic tool in managing intestinal inflammatory disorders.

Finally, a recent report suggested a potential application of bovine milk exosomes in the management of malnutrition. Exosomes were administered to mice fed a 1% protein diet and were shown to improve defective intestinal epithelial permeability and architecture induced by malnutrition [140]. Further studies are warranted to evaluate the therapeutic value of bovine milk exosomes in malnourished individuals.

### 4.2. Bone and Muscle Metabolism

Bone remodeling is a dynamic process characterized by consecutive cycles of bone resorption and formation regulated by specialized cells, namely osteoclasts and osteoblasts [141]. While the attachment of osteoclasts to the bone surface leads to bone resorption mediated by acidification and proteolysis processes, the osteoblasts are responsible for bone formation mediated by tightly regulated processes of matrix production and bone mineralization [141]. Bone remodeling is controlled at the systemic level by hormones responsible for maintaining calcium homeostasis, mainly parathyroid hormone and vitamin D metabolites [141]. Milk is not only a rich source of calcium and vitamin D3 but also contains different minerals and proteins, such as lactoferrin and whey proteins that are involved in bone remodeling processes [142,143,144,145]. Although different mechanisms support a positive role of milk intake in bone health and the prevention of bone fractures, some studies have failed to prove these effects [146]. Nonetheless, milk consists of a complex matrix, and the health effects of individual components might not be completely extrapolated [147]. In this sense, some authors have focused on milk extracellular vesicles to identify milk bioactive compounds with beneficial effects on bone formation. In particular, Oliveira et al. orally administered two different concentrations of bovine milk exosomes (4.7 × 10^6^/mL and 14.3 × 10^6^/mL) to mice for seven weeks observing that treated mice displayed increased osteocytes number and woven bone formation compared to control (PBS) [148]. Furthermore, exposing human mesenchymal stem cells (hMSCs) to bovine milk exosomes led to increased osteoblast differentiation. In an ensuing study, Oliveira et al. showed that milk exosomes treatment on murine bone marrow cells induced an increase in osteoclast differentiation along with an inhibition of the osteoclast activity [149]. Recent research conducted by Go et al. studied the addition of bovine milk exosomes to human osteoblastic Saos-2 cells in vitro, observing an increase in Saos-2 cells proliferation in a time-dependent and dose-dependent manner while promoting the expression of the osteoblast transcription factors RUNX2 and Osterix [150]. Moreover, treatment of Saos-2 and MC3T3-E1 pre-osteoblastic cells with milk exosomes increased alkaline phosphatase (ALP) and osteocalcin (OCN) levels, which are involved in extracellular matrix synthesis processes [150]. Finally, the authors administered 50 mg/kg/day of exosomes to rats for 14 days and reported that the treatment was associated with significantly increased bone mineral density in trabecular and cortical tibiae [150]. In the light of these studies, bovine milk exosomes could constitute food components with the potential to influence different aspects of bone remodeling.

The aforementioned effects of bovine milk exosomes in osteogenesis lay the groundwork for the study of their potential application in settings of bone loss and osteoporosis. Oliveira et al. explored the osteoprotective properties of bovine milk exosomes in obese and ovariectomized mice. The treatment with bovine milk exosomes contributed to a reduced RANKL/OPG ratio in mice fed a high carbohydrate diet, which denotes decreased osteoclast differentiation and activity. In ovariectomized mice, the administration of bovine milk exosomes prevented the loss of mechanical resistance and improved several features of femur microarchitecture [151]. In addition, the administration of exosomes was also associated with decreased number and activity of osteoclast cells in ovariectomized mice by reducing the local and systemic RANKL/OPG ratio [151]. Other authors have explored the use of bovine milk exosomes to improve bone health in the context of osteoporosis. Yun et al. treated MC3T3-E1 and RAW 264.7 cells with bovine colostrum exosomes. No cytotoxicity was evident in MC3T3-E1 cells exposed up to 500 ng/mL of exosomes, while 100 ng/mL was enough to enhance cell proliferation. Regarding the RAW 264.7 cells assay, exosomes were shown to inhibit osteoclast differentiation after exposure to RANKL and macrophage-colony-stimulating factor [98]. Furthermore, 8-week treatment with colostrum exosomes before the onset of steroid-induced osteoporosis exerted a positive effect on the femoral bone volume and bone mass density [98]. Thus, bovine milk exosomes appear to be a promising element to explore the prevention of bone loss and osteoporotic processes.

Milk intake has been associated with improved linear growth in various observational and interventional studies [152,153,154]. In the same line, milk allergy has been linked to a lower final height in adulthood [155]. Not only has milk intake been associated with linear growth, but it also may be beneficial for malnourished children undergoing catch-growth [145]. Importantly, relationships between growth and milk intake can be subjected to reverse causality and residual confounding. Notwithstanding this fact, the potential effects of milk on stature growth have been attributed to different milk components, such as proteins, minerals, and prebiotic carbohydrates [156]. These effects are usually proposed to be mediated through the stimulation of IGF-1 synthesis, which constitutes a major regulator of growth and bone elongation [157,158,159]. Interestingly, some authors have proposed that breastfeeding is linked to higher IGF-1 levels later in life, which implies a programming effect of breastmilk on linear growth patterns [160]. MiRNAs abundantly expressed in milk exosomes, namely miRNA-148a and miRNA-29, have been hypothesized to induce IGF-1-mediated growth [118,161].

**Figure 4:**
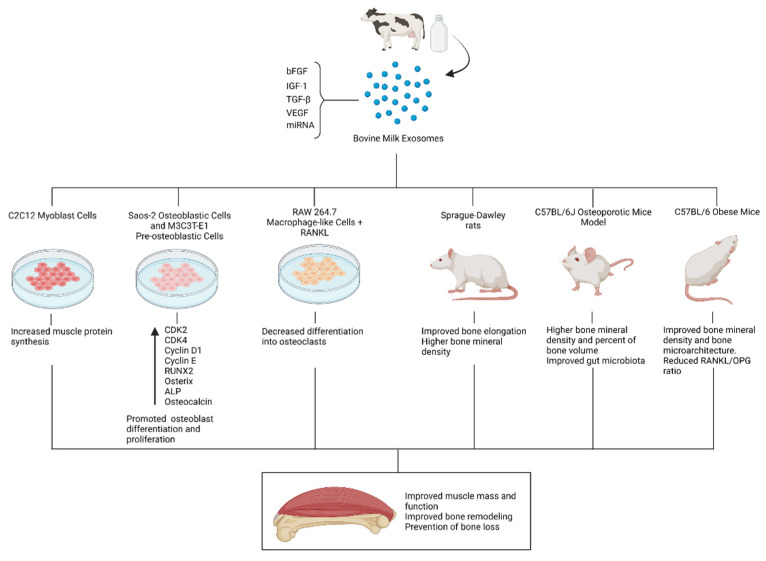
Potential beneficial effects of milk exosomes on the musculoskeletal system.

Bone and muscle constitute a functional unit [162]. Not only adequate bone remodeling but also an appropriate muscle function are desirable to support a healthy musculoskeletal status. Nutritional interventions targeting both bone mass and muscle function may have profound implications for mobility and quality of life. In this regard, milk contains valuable proteins with important anabolic effects in terms of muscle protein synthesis [163]. Nonetheless, different milk components might contribute to these anabolic effects. On this subject, Mobley et al. explored the effects that exosomes isolated from the whey fraction of bovine milk had on features of muscle anabolism in vitro. Seeding C2C12 myoblast cells with milk exosomes led to a significant increase in muscle protein synthesis 12 and 24 h after treatment. Although mTORC1 was not affected 6–24 h after the treatment, a short-term activation could not be discarded [164]. The following study evaluated the administration of bovine milk exosomes to C57BL/6 mice in vivo, observing only modest effects on gene expression and amino acid content in treated animals with no relevant impact on muscle strength [165]. These results were in line with those obtained by Parry et al., who documented that a bovine milk exosome-depleted diet elicited anabolic and transcriptomic effects in rat muscle, whereas an exosome-sufficient diet was not associated with any anabolic outcome [166]. Overall, in vitro results suggested a potential role for bovine whey exosomes in muscle anabolism, but evidence from in vivo studies is conflictive. Differences in tissue distribution patterns of milk exosomes might be responsible for these heterogeneous results [165,166], yet cross-communication between different organs should not be discarded [33]. Promising in vitro results should not be eclipsed by these discrepancies, and further research on this subject is warranted to elucidate the potential role of milk exosomes in muscle anabolism. A summary of the preclinical evidence on the potential beneficial effects of bovine milk exosomes on musculoskeletal health is illustrated in Figure 4.

### 4.3. Immunity

Breast milk is a dynamic composition of nutrients and bioactive factors that changes throughout lactation according to the needs of the developing infant [167]. A solid body of literature supports that breastfeeding confers protection against infections, allergic diseases, and inflammatory processes in infants [168,169,170,171]. In this sense, a wide number of breast milk components present immunoregulatory properties and might contribute to these effects, including immunoglobulins, oligosaccharides, glycoproteins, living cells from the mother, and probiotic microorganisms, among others [167]. Exosomes are among the pool of bioactive compounds with immunoregulatory features identified in milk. Admyre et al. reported the presence of exosome-like vesicles in human breast milk, which, when incubated with peripheral blood mononuclear cells (PBMC), led to inhibited production of IL-2, IFN-γ, and TNF-α along with increased production of IL-5 [61]. These effects were proposed to be mediated by the regulation of T-lymphocytes response [61]. Authors postulated that these effects might contribute to the benefits of breast milk in the development of the infant’s immune system. Kosaka et al. examined milk exosomes cargos observing that these vesicles contained miRNAs with the potential to induce B-lymphocytes differentiation, namely miR-181 and miR-155 [172]. Following an in-depth analysis of human milk exosomal miRNA reported that immune-related miRNAs are present in high quantities in this medium with low variation among healthy individuals [92]. Nowadays, it is well-accepted that milk exosomes carry a notable number of miRNAs with potential immunomodulatory effects [173]. The potential immunomodulatory properties of milk exosomes might be at least partially responsible for the already discussed benefits on intestinal health and other health outcomes [174].

Interestingly, not only human breast milk but also milk from different mammals display a high miRNA content, and exosome immune-related miRNA profiles are similar across species [175]. In particular, miRNAs members of the let-7 family and miR-148a were shown to be highly conserved in milk exosome samples obtained from a wide variety of different mammals and have been implicated in several aspects of the immune function [90,120,176,177]. More precisely, members of the miR-148/152 family were demonstrated to inhibit the TLR-mediated expression of MHC II and cytokine production by targeting CaMKIIα in dendritic cells [178]. MiRNA-148a has also been linked to the regulation and function of lymphocytes B and T and might play a role in the prevention of inflammatory and autoimmune disorders [179]. Similarly, members of the let-7 miRNA family play an important role in regulating TLR4 signaling, contributing to host defense responses in settings of infection [180]. Furthermore, let-7 has been linked to processes of macrophage activation and modulation of the adaptative immune response [180]. Recently, immune-related miRNAs, such as miR-181a, miR-26a, and miR-191, were found to be among the most abundantly expressed in colostrum and mature bovine milk exosomes [98]. Hence, it can be inferred that miRNAs present in milk exosomes from different species might serve the purpose of contributing to the development of the infant’s immune system.

Different cargos might also contribute to the immunomodulatory properties of milk exosomes. Regarding proteins, Benmoussa et al. analyzed possible pathways impacted by proteins contained in milk exosomes observing that identified proteins were associated with different immunity pathways related to neutrophil degranulation and the innate immune system, among other aspects [181]. Specifically, Pieters et al. observed that transforming growth factor-β (TGF-β) was consistently expressed in exosomes isolated from commercial semi-skimmed cow milk [68]. TGF-β cytokine is crucial for the differentiation of Th17 cells [182]. To assess exosomes activity, authors co-incubated murine spleen T-cells with a Th17 differentiation cocktail replacing TGF-β with milk exosomes, confirming the differentiation of Th17 cells, which was repressed by the blockade of TGF-β using monoclonal antibodies [68]. These results aligned with a study evaluating similar settings [183]. Likewise, another study reported TGF-β1 as a cargo of both cow and human milk exosomes [69]. Curiously, one report on proteomic and functional enrichment analysis of bovine milk exosomes suggested that immune-related proteins are more abundant in colostrum compared to mature milk [64]. Nonetheless, a recent proteomic analysis of late-stage lactation bovine milk exosomes revealed a large number of proteins that were mostly related to metabolism and immune system pathways [184]. These findings underscore that not only nucleic acids but also proteins might contribute to the immunomodulatory properties of exosomes isolated from different types of milk. Finally, as commented, data regarding the lipid content of exosomes and its potential effects on immune outcomes are limited and might constitute an interesting field to explore.

**Figure 5:**
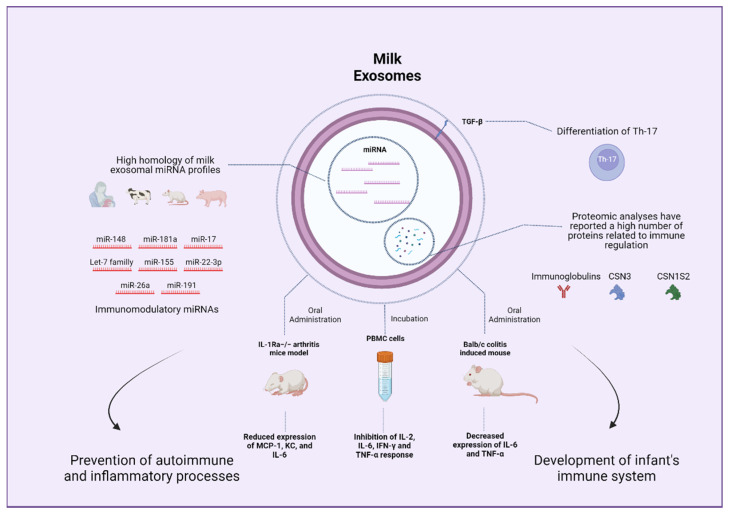
Potential beneficial effects of milk exosomes on the immune system.

Research on the regulatory effects of milk exosomes in different immune processes has been reported. In this regard, different macrophage cell lines have been consistently shown to take up milk exosomes. A recent study observed that the treatment of RAW264.7 macrophages with bovine exosomes before LPS stimulation contributed to a decreased inflammatory response and a downregulated cytokine secretion mediated by inhibited NF-κB pathway [185]. Other studies conducted in RAW264.7 cells have demonstrated that bovine milk exosomes stimulate macrophages proliferation without stimulating nitric oxide or proinflammatory cytokines production while promoting the expression of proteins involved in cell cycle and proliferation [186]. Furthermore, bovine milk exosomes were shown to exert a protective effect against the cytotoxic action of cisplatin, a chemotherapeutic drug, in RAW264.7 cells [186]. Regarding immune effects on different cells, Arntz et al. pre-incubated mice splenocytes with different concentrations of exosomes (20 and 200 μg/mL) to later stimulate the cells with LPS. Exosomes treatment led to a pronounced reduction in proinflammatory markers TNF-α and monocyte chemoattractant protein-1 (MCP-1) in response to the LPS insult compared to control [183]. Furthermore, IL-1Ra knockout mice, which display symptoms of polyarticular arthritis, were administered different doses of bovine milk exosomes. Mice treated with 1200 μg/mL of exosomes showed a considerable delay in arthritis onset and reduced arthritis symptoms, which implies a potential role of bovine milk exosomes in autoimmune and inflammatory diseases [183]. Interestingly, milk exosomes have been suggested to confer protection against HIV infection. Milk exosomes were shown to express soluble mucin 1 (MUC1) and incubation of monocyte-derived dendritic cells (MDDCs) with exosomes was observed to protect against HIV-1 infection in vitro [187]. Moreover, milk exosomes were taken up by MDDCs within 4 h and prevented the transfer of HIV-1 from MDDCs to CD4^+^ T cells, whereas plasma-derived exosomes did not exhibit any of these effects [187]. Overall, the literature supports an immunomodulatory role of milk exosomes as diet components that may impact an infant’s development and different immune processes (Figure 5).

### 4.4. Microbiota

EVs have been proposed to facilitate a cross-talk process between the host and the microbiome [188]. Milk exosome microRNAs display unique distribution profiles and are accumulated in the intestine, among other organs [87]. Importantly, a fraction of dietary exosomes and their cargos are not absorbed and can reach further sections of the intestine [87]. Small compounds such as plant-derived nanoparticles, which contain proteins and miRNAs [189], have been observed to be taken up by gut bacteria and elicit changes in the gut microbiota composition [190]. Hence, it seems reasonable to conceive a potential interaction between dietary milk exosomes and the gut microbiota.

Yu et al. characterized three EVs from different sources, namely pasteurized bovine milk, coconut water, and adipose-derived stem cells, and compared their uptake by common representative gut bacteria and analyzed their effects on bacterial growth [191]. The authors observed that exosomes and exosome-like nanoparticles were capable of supporting bacterial growth and modulating gene expression in vitro. Specifically, milk exosomes promoted the growth of *E. coli* and *L. plantarum* commensal strains [191]. Zhou et al. evaluated the long-term administration of either bovine milk exosome-sufficient or bovine milk exosome-depleted diets to C57BL/6 mice and analyzed bacterial communities ex vivo. Three phyla, seven families, and 52 operational taxonomic units were differentially abundant between mice allocated to the exosomes-sufficient diet compared to the exosomes-depleted diet group [116]. An ensuing study conducted by Tong et al. evaluated the impact that an eight-week duration treatment with different bovine milk exosomes concentrations had on features of gut microbiota and intestinal immunity in C57BL/6 mice [115]. Authors reported that milk exosomes led to a higher relative abundance of Clostridiaceae, Ruminococcaceae, and Lachnospiraceae compared to control (PBS) [115]. Furthermore, SCFAs production was enhanced in treated animals displaying increased levels of acetate, propionate, and butyrate, and the administration of moderate quantities of milk exosomes correlated to increased levels of IgA and sIgA in mice’s intestine [115]. Notably, a recent study conducted by the same authors documented that the administration of milk exosomes to a DSS-induced colitis mice model restored gut bacteria relative abundance near the levels displayed by control mice [139]. Finally, another recent report confirmed a protective effect of milk exosomes and a miRNA-sufficient diet in the severity of *C. difficile* infection in C57BL/6 mice when compared to the administration of a milk exosomes and miRNA depleted diet [192]. As commented, milk exosomes elicit immunomodulatory effects and might convey beneficial effects on intestinal health. Hence, the effects of milk exosomes on gut microbiota should be considered within this context. In the light of these results, milk exosomes constitute valuable milk bioactive compounds that might influence wide aspects of intestinal health and microbiota.

### 4.5. Neurodevelopment

Breastfeeding has been consistently associated with long-term positive cognitive outcomes [193]. Accordingly, epidemiological studies have linked breastfeeding to higher intelligence quotient scores; and improved cognitive, language, and motor skills later in life, compared to formula feeding [193]. Different breast milk components might contribute to brain development and cognitive function [194,195]. Preclinical evidence suggests that milk exosomes and their cargos cross the brain-blood barrier and accumulate in the brain following oral intake [87,196]. On this subject, the depletion of dietary bovine milk exosomes was reported to impair cognitive performance in C57BL/6 mice compared to an exosome-sufficient diet [197]. Mice displayed improved spatial learning and memory when fed the exosome-sufficient diet compared to the exosome-depleted diet [197]. The depletion of bovine milk exosomes and their RNA cargos from the diet was linked to increased hepatic purine metabolites in mice and higher plasma and urine excretion of purine metabolites in humans, which led the authors to speculate that the regulation of purine metabolism by milk exosomes might mediate their positive effects at cognitive level [198]. In this context, it could be proposed that exosomes take part in the neurodevelopmental properties of breast milk. Accordingly, numerous miRNAs contained in milk exosomes, such as miR-148a, miR-141-3p, miR-375, and miR-107, might be involved in nervous system pathways and brain development [199,200]. However, sphingomyelin and other phospholipids present in milk exosomes have been demonstrated to promote neurodevelopment in preclinical research and clinical studies [196]. Furthermore, miRNAs targeting glycosphingolipid biosynthesis pathways were shown to be highly expressed in preterm breast milk, which has a major role in brain development [201]. As commented, different mechanisms support a potential role of milk exosomes in infant neurodevelopment and further research is warranted.

## 5. Limitations

Milk exosomes are promising ingredients, which can act as drug delivery vehicles, and contain bioactive cargos with the potential to influence different health outcomes. However, some studies have cast doubts on their capacity to elicit significant effects following oral intake. Several preclinical and clinical studies have failed to show a positive association between milk intake and serum miRNA levels [202,203]. Nonetheless, these studies did not specifically focus on exosomal miRNA and aspects related to sample conservation and the use of miRNA reference sequences might compromise their results [103]. In the same line, studies in favor of milk miRNA availability in humans (i.e., [204]) have been subjected to criticism due to methodological concerns [205,206]. On the contrary, preclinical research on the assimilation of exosomal miRNA has yielded more promising results [87,160]. The effects of milk exosomes are at least partially attributed to their nucleic acid content. Yet, miRNAs not only must survive industrial processing and storage conditions but also persist after oral intake, being absorbed, and reaching the targeting cells in sufficient amounts to exert a significant effect [103]. There is a need to evaluate if the promising results observed in animal models can be extrapolated to clinical research. Clinical studies evaluating the effect that the depletion of milk exosomes compared to exosome-sufficient milk has on serum miRNA profile are lacking and might shed light on milk exosome bioavailability. In the same fashion, studies conducted on human organoids would provide insights into exosomes bioactivity [174]. Finally, future studies should consider the integrity of isolated exosomes when assessing their bioavailability after oral intake.

Isolation protocols and characterization methods should be harmonized to ensure the replicability of milk exosomes studies [207,208]. Furthermore, isolation methods should be optimized to enable large-scale production without compromising exosome bioactivity in compliance with good manufacturing practices [88]. The purification of exosomes is important to avoid other milk constituents or contaminants which might jeopardize their quality in preclinical research [174,208], and rigorous compliance with the MISEV guidelines is necessary to attribute milk exosomes effects reported in exosomes studies [209].

Some reviews have raised concerns about the possible deleterious effects of milk exosome intake. Accordingly, milk exosomes would contribute to the development of diabetes, atherosclerosis, osteoporosis, Parkinson’s disease, diverse types of cancer, and even all-cause mortality mediated by different mechanisms such as over-activation of the mechanistic target of rapamycin complex 1 (mTORC1), among others [210,211,212,213,214]. These effects would not be attributed to fermented milk because fermented milk displays reduced exosome miRNA, branched-chain amino acids, and protein content due to bacterial activity [212]. On the other hand, a recent paper by one of these authors extensively disclosed the beneficial effects of milk exosomes on infant intestinal health, β-cells maintenance, bone homeostasis, adipogenesis, and neurodevelopment [118]. These contradictory data suggest that the consumption of non-fermented milk leads to strikingly opposed effects depending on the consumer’s age. However, this argument is not supported by epidemiological studies evaluating milk or dairy products intakes, and some of the proposed pathways are independent of the consumer’s age. According to the FAO, there is no solid evidence regarding the deleterious effects of milk and normal intakes of dairy products on any health outcome [215].

## 6. Future Perspectives

Due to their high biocompatibility and stability, milk exosomes have been proposed as promising vehicles for the delivery of hydrophilic and lipophilic bioactive compounds [216]. In contraposition to artificial delivery systems, these formulations might present lower immunogenicity, superior bioavailability, increased capacity to cross biological barriers, and the ability to confer protection against harsh conditions, thus improving the biological effects of delivered molecules while reducing their potential toxicity [32,59,217,218]. Specifically, the use of milk exosomes to deliver epicatechin gallate (ECG), a naturally occurring polyphenolic compound with neuroprotective properties, was shown to improve its antioxidative and antiapoptotic effects while inhibiting autophagy in a rotenone-induced model of Parkinson’s disease in vitro, compared to the incubation with free ECG [217]. Similarly, milk exosomes have been explored to improve the bioavailability of different natural compounds with positive results, including curcumin and anthocyanidins [219,220]. Curcumin is a bioactive polyphenol with anti-inflammatory and antioxidative properties, which has been proposed to exert beneficial effects on obesity and protect against neural damage, among other functions [221,222,223]. In the same line, anthocyanidins derived from bilberries have been observed to inhibit T-cell cytokine signaling and IFN-γ transduction in settings of ulcerative colitis [224]. However, these compounds present poor bioavailability after oral intake. Modulating the bioavailability of these compounds is crucial for improving their use as nutraceutical ingredients [225,226]. Milk exosomes represent promising natural delivery vehicles that might enhance the bioavailability of bioactive ingredients contained in these formulations.

To establish milk exosomes as ingredients and delivery vehicles, it is mandatory to overcome some limitations of current isolation and characterization methods, mainly those related to the inability to produce inexpensive and large-scale quantities of milk exosomes [40]. For instance, novel microfluidics-based techniques yield high purity exosomes and are automatable but lack scalability and high sample capacity [59,218]. Optimization of current ultracentrifugation and filtration protocols (i.e., TFF), as well as the use of mixed approaches, including combinations of different characterization techniques, might be best suited for the large-scale production of high-quality milk exosomes [40,227].

Besides the already-mentioned cargos, milk exosomes might contain or be associated with different compounds with potential beneficial effects, such as antibodies, functional lipids, or oligosaccharides. As recently reported by He et al., several milk oligosaccharides, including 2-fucosyllactose with potential prebiotic and immunomodulatory properties [228], were isolated from human colostrum and mature milk exosomes [229]. The in vivo administration of these encapsulated oligosaccharides was shown to modulate macrophages activation in vitro, attenuate adherent-invasive *Escherichia coli* infection in dextran sodium sulfate mice models in vivo, and prevent LPS-induced inflammation and intestinal damage in these animals [229].

Beyond miRNAs, different nucleic acids contained in milk exosomes have been suggested to play roles in different health outcomes. Long noncoding RNAs (lncRNAs) are transcripts of more than 200 nucleotides that do not translate into proteins [230]. LncRNAs identified in human breast milk exosomes were proposed to be implicated in infant metabolism, neonatal immunity, and development [231]. Not only lncRNAs but also different nucleic acids, such as circular RNAs (circRNAs), covalently closed RNA molecules capable of modulating miRNAs activity, have been reported in milk exosomes [232]. CircRNAs identified in bovine milk-derived exosomes were linked to genes involved in the cytoplasm, endoplasmic reticulum, transport, and transcription factors [232]. In an ensuing study, Zeng et al. performed functional analysis on circRNAs and lncRNAs identified in pig milk-derived exosomes predicting that these would be involved in features of intestinal barrier status, including adherence junction, tight junction, and inflammation [230]. Although further research is needed, some authors have postulated potential roles for milk exosome-derived circRNAs in developing intestine and gut microbiota during early life [233].

Proteomic analyses have concluded that various immunoglobulin components are contained in bovine milk exosomes [26,64]. Interestingly, Betker et al. observed that the “neonatal” Fc receptor (FcRn), a receptor expressed throughout life and involved in IgG binding and transport, participates in milk exosomes uptake [114]. Furthermore, the authors demonstrated the presence of substantial amounts of both bound and unbound bovine IgG in milk exosome isolates [114]. The presence of antibodies among exosomal cargos further supports their immunomodulatory properties.

As we review throughout the present manuscript, milk exosomes have been linked to the prevention of disorders linked to perinatal health such as NEC [134] and might modulate wide aspects of immunity and development, but studies analyzing infant formulae have reported very low quantities of exosomal miRNAs in these formulations [109,234]. Several authors have considered the addition of miRNA-containing milk exosomes to infant formulae as a strategy to reduce the prevalence of intestinal disorders [134,235]. Similarly, the addition of miRNA cargos related to the development of the immune system, such as miR-155, might contribute to the prevention of atopic disease in infants [29]. Other miRNA cargos linked to neurodevelopment, lipid, and glucose metabolism might support adequate infant growth and development [95]. Overall, the benefits of breastfeeding are well-established in the literature, and the natural presence of milk exosomes in breast milk provides a new dimension to perinatal nutrition. Infant formulae might benefit from the addition of these natural compounds as bioactive ingredients. Furthermore, infants and adults might take advantage of the potential positive effects of milk exosomes in areas such as muscle strength, osteoporosis processes, immunity, and intestinal health.

## 7. Conclusions

The presence of exosomes in breast milk might represent a shift in the paradigm in human nutrition and constitutes a burgeoning field of research. Milk exosomes contain a variety of bioactive cargos, which have been reported to exert beneficial effects at multiple levels, including but not limited to immunity, intestinal and musculoskeletal health, gut microbiota, growth, and development. Further research is necessary to extrapolate to clinical studies the promising results observed in preclinical studies.
